# Community-engaged passive tick surveillance highlights the diversity of ticks and tick-borne pathogens in Illinois

**DOI:** 10.1371/journal.pone.0355935

**Published:** 2026-08-13

**Authors:** Holly C. Tuten, Lance E. Jones, Jannatul Promee, Chang-Hyun Kim, Chris M. Stone

**Affiliations:** Illinois Natural History Survey, Prairie Research Institute, University of Illinois Urbana-Champaign, Champaign, Illinois, United States of America; University of Kentucky College of Medicine, UNITED STATES OF AMERICA

## Abstract

The continuing increase in incidence of tick-borne illnesses in the United States necessitates expanded surveillance, especially in areas like Illinois experiencing range expansion of key vectors such as *Ixodes scapularis* and *Amblyomma americanum*. We report on the initial years (2018–2023) of a community-engaged passive tick surveillance program in Illinois designed to complement active collections by casting a wide net for tick species, tick-host encounters, and emerging pathogens. Participants submitted eight tick species, dominated by *A. americanum*, *I. scapularis*, and *D. variabilis*. Submissions, primarily adults, occurred almost year-round (except February), and were most often associated with humans, dogs, and deer. We used a microfluidic system and sequencing to test a subset of the submitted ticks. Six tick-borne pathogens were detected, including *Borrelia burgdorferi* s.s. (14% prevalence in adult *I. scapularis*), *B. miyamotoi* (4.7% in adult *I. scapularis*), and *Anaplasma phagocytophilum* (1.2% in *I. scapularis* as well as 1 out of 7 tested *Dermacentor albipictus*). We also documented spotted fever group *Rickettsia* species, such as *R. amblyommatis* (53% prevalence in nymphal *A. americanum*), *R. montanensis* (2.1% in *D. variabilis*), and *Candidatus* R. andeanae (in 2 out of 3 *A. maculatum*). These efforts helped document the year-round risk of tick exposure and expanded our knowledge of the diversity of ticks and circulating pathogens in Illinois. We believe the combination of a sustainable, low-cost passive surveillance program coupled with multi-pathogen assays can serve a useful function as complementary surveillance for emerging pathogens and changing tick distributions.

## Introduction

Cases of tick-borne illness in the United States have continued to rise in recent decades, and are now the most common vector-borne infections in this region [1]. To a large extent, the rise in cases of tick-borne diseases is thought to be associated with the ongoing geographic spread of some of the tick species involved in transmission of pathogens to humans [2]. This includes for instance the ongoing range expansion of *Amblyomma americanum* [3,4] and the expansion of *Ixodes scapularis* from foci in the Northeastern U.S. and the Upper Midwest, including into states such as Illinois [5,6].

Along with the spread of these vectors and increases in cases of tick-borne infections such as Lyme disease or spotted fever group Rickettsioses (SFGR) in recent decades, we have observed the introduction of the invasive tick species *Haemaphysalis longicornis* into the U.S. [7]. Although the veterinary and public health consequences of this invasion are still coming into focus, this species has been associated with a variety of tick-borne pathogens in its native range [8]. Additionally, in the U.S. we have seen the continued emergence and recognition of new tick-borne pathogens that cause human illness, such as *Rickettsia parkeri*, *Borrelia miyamotoi*, Heartland virus, and others [9]; these emerging pathogens have likewise been detected in Illinois [10–12].

Due to these growing threats related to the spread and emergence of ticks and tick-borne pathogens, an urgent need for increased tick and tick-borne pathogen surveillance efforts has been recognized [13], in order to better track changing distribution patterns, provide information on local presence of ticks and tick-borne pathogens to physicians and medical providers, or to serve as a baseline and information source for tick-bite prevention methods [14]. This need for surveillance is particularly relevant in areas at the current edges of tick range distributions, where systematic historical surveillance efforts may not have been established. This has been recognized for Illinois [15], which began systematic active surveillance efforts in 2019 (e.g., [12,16].

Here, we report on an ongoing community-engaged passive tick surveillance program that was developed to complement ongoing statewide active tick surveillance efforts for Illinois. Passive or citizen science based tick surveillance programs have been recognized as being able to supplement environmental tick collections in several useful ways, for instance by providing different insights into people’s risk of exposure to tick bites than would be obtainable through dragging or trap-based collections, although interpretation of results needs to be done with care [17]. Additionally, passive collections can supplement tick dragging by increasing sampling coverage over a larger geographic area than is obtainable with active surveillance, which can facilitate detection of overlooked hotspots of infection or serve as an early warning system for invasive tick and pathogen species. Discoveries of ticks or pathogens in areas where they were not thought to occur could then inform targeted active surveillance and improve resolution and cost-effectiveness of tick-borne disease prevention efforts.

We aimed to develop our passive tick collections as a sustainable, low-cost effort in collaboration with a broad community of stakeholders. The objectives were to cast a wider net than active collections could achieve in terms of the diversity of tick species collected, to document tick-host encounters, and to allow for the possibility of detection of rare or emerging pathogens or potential hotspots by using a multi-pathogen testing panel. Here, we report on the distribution of tick specimens and their associated pathogens by county, based on submissions from the initial years (2018–2023) that we provided tick identifications for the public.

## Methods

### Tick submissions

Submissions by the public were encouraged by listing the program as a tick identification service on the Illinois Natural History Survey Medical Entomology Laboratory website (https://medical-entomology.inhs.illinois.edu/research/free-tick-identifications/) with a downloadable form with instructions. This was done starting in 2019, but a small number of tick samples were submitted through individual contacts in 2018 and are included here. The program was further promoted via social media and public outreach events. The form required participants to provide the county of residence, the date of tick encounter (if not known, the week, month and year), and the most likely encounter location (state, county, zip code). Additionally, participants were asked to indicate whether the tick was attached, the date and place of removal, and if so, whether they had travelled outside of their county of residence in the past ten days. If the host of the tick (human, or other species of vertebrate animal) was indicated, this was recorded as well. Participants submitted ticks in a Ziploc bag, with either a handful of fresh green leaves if alive or a cotton ball damp with rubbing alcohol if dead, or sometimes just in a Ziploc bag. The condition of submitted ticks ranged from good to desiccated or damaged. Instructions for shipping ticks included an option to send live ticks on the assumption this could lead to better DNA/RNA quality and potentially offer the ability to screen for tick-borne viruses in the future. Submissions came from the general public, as well as organizations including the Forest Preserve District of DuPage County, the Chicago Park District, and the Illinois Lyme Association. Submissions varied from single ticks to multiple, and some individuals or groups submitted or dropped off ticks multiple times. Submitted ticks were frozen upon receipt and those from within the state were identified to species using light microscopy and dichotomous keys [18–23]. If samples were not in a condition where this was possible (e.g., due to missing mouth parts or damage), they were identified to genus only. Ticks were subsequently transferred to vials of 85% ethanol and kept at 4 °C.

### DNA extractions and pathogen testing

Only a subset of ticks were tested due to limited funding, available in 2022 and 2023, with the greatest focus being on the three tick species of primary human health concern (*I. scapularis*, *D. variabilis*, and *A. americanum*), while also including some of the other submitted species as well. Tested samples included a majority of the submissions from 2023 (104 individuals or pools), and smaller numbers of individuals or pools from 2018–2022 (5, 16, 19, 7, 28, respectively). Beyond ensuring a level of representation among program years and species, we did not apply specific criteria to selection for testing. Any samples where the county of origin was unclear or where travel was indicated for attached ticks (18 in total) were subsequently removed from the analysis and not included in the results presented here. Ticks that were selected for pathogen testing underwent the following DNA extraction procedure. Individual, or in the case of nymphal *A. americanum*, pools of up to 5 specimens were homogenized in a bead tube containing 5 stainless steel beads, 200 µl buffer BE, 80 µl buffer MG (or buffer LBP) (Takara Bio USA, San Jose, CA) and 20 µl liquid proteinase K (Thermo Fisher Scientific, Waltham, MA) in a Qiagen Tissue Lyser II at 30 cycles / second for 20 minutes. For the first 75 samples, DNA was extracted manually using the Takara NucleoSpin DNA RapidLyse kit (Takara Bio USA, San Jose, CA) following manufacturer’s instructions and for the remaining 105 samples, KingFisher Virus/Pathogen Ultra kit was used to isolate DNA automatically using a KingFisher Apex platform (Thermo Fisher Scientific, Waltham, MA). DNA concentration of the elute was measured using a Qubit dsDNA HS assay (Invitrogen, Waltham, MA).

We used the Fluidigm Biomark high-throughput microfluidic system (Standard BioTools, San Francisco, CA) to perform parallel real time PCR assays on tick samples on a suite of tick-borne pathogens, adapted from a similar set of previously published assays [24–26]. The primers and probes used for each specific pathogen assay are listed in Table 1. Performance of the microfluidics assays was performed at the Roy J. Carver Biotechnology Center at the University of Illinois Urbana-Champaign. Tests were run in triplicate, and included positive (plasmids containing the relevant PCR amplicons) and negative controls. Ticks were tested using three separate Fluidigm runs. On tests 2 and 3, we experimented with including an additional assay for *B. miyamotoi*, based on the real time PCR (rt-PCR) assays of Graham et al. [27]. Due to a lack of amplification of positive controls on the Fluidigm runs for both sets of primers targeting the *glpQ* gene for *B. miyamotoi*, these were repeated subsequently through standalone rt-PCR performed on a Quantstudio 5 (Applied Biosystems, Waltham, MA), as well as for *B. burgdorferi s.s.* for additional confirmation, using the primer sets of Graham et al. [27]. Where there were inconsistencies between *Borellia* sp., the Fluidigm assays, and the rt-PCR results, we followed up with Sanger sequencing and only considered the test positive if confirmed by a BLAST comparison (Accession numbers: PZ482904−05). In the case of *R. amblyommatis*, we noticed cross reactivity between this assay and that of *R. montanensis*. For a subset of *A. americanum*, and for all specimens of other tick species that were positive for either pathogen on the Fluidigm assay, we used Sanger sequencing and GenBank BLAST searches to clarify the rickettsial species present (Accession numbers: PZ482906−16). Because some *A. americanum* nymphs were tested in pools, we calculated prevalence estimates and confidence intervals for all pathogens of interest using maximum likelihood with the PoolTestR package in R [28].

**Table 1:** Overview of primers and probes used in this study for pathogen assays.

Species	Target gene	Name	Sequence	Reference
*Anaplasma phagocytophilum*	*msp2*	An_ph_msp2_F	GCTATGGAAGGCAGTGTTGG	[24]
		An_ph_msp2_R	GTCTTGAAGCGCTCGTAACC	
		An_ph_msp2_P	FAM-AATCTCAAGCTCAACCCTGGCACCAC-BHQ1	
*Anaplasma spp.*	*groEL*	F-Anap	GCGAGCATAATTACTCAGAG	[29]
		R-Anap	AGTATGGAGCATGTAGTAG	
		Cy5	FAM-CCACCTTATCATTACACTGAGACG-MGBNFQ	
*Babesia microti*	*CCTeta*	Bab_mi_CCTEta_F	ACAATGGATTTTCCCCAGCAAAA	[24]
		Bab_mi_CCTEta_R	GCGACATTTCGGCAACTTATATA	
		Bab_mi_CCTEta_P	FAM-TACTCTGGTGCAATGAGCGTATGGGTA-MGBNFQ	
*Bartonella spp.*	*ssrA*	ssrA-F	GCTATGGTAATAAATGGACAATGAAATAA	[30]
		ssrA-R	GCTTCTGTTGCCAGGTG	
		ssrA-P	FAM-ACCCCGCTTAAACCTGCGACG-MGBNFQ	
*Borrelia burgdorferi s.s.*	*oppA2*	Bbss_F	AATTTTTGGTTCCATACCC	[27]
		Bbss/Bmayo_R	CTGTCAATAGCAAGAGTTAA	
		Bbss_P	VIC-CGTTCAATACACACATCAAACCACT-MGBNFQ	
*Borrelia burgdorferi s.s.*	*rpoB*	Bo_bu_rpoB_F	GCTTACTCACAAAAGGCGTCTT	[24]
		Bo_bu_rpoB_R	GCACATCTCTTACTTCAAATCCT	
		Bo_bu_rpoB_P	FAM-AATGCTCTTGGACCAGGAGGACTTTCA-BHQ1	
*Borrelia miyamotoi*	*glpQ*	B_miya_glpQ_F	CACGACCCAGAAATTGACACA	[24]
		B_miya_glpQ_R	GTGTGAAGTCAGTGGCGTAAT	
		B_miya_glpQ_P	FAM-TCGTCCGTTTTCTCTAGCTCGATTGGG-BHQ1	
*Borrelia miyamotoi*	*glpQ*	C46	GACCCAGAAATTGACACAACCACAA	[27]
		C47	TGATTTAAGTTCAGTTAGTGTGAAGTCAGT	
		C48	VIC-CAATCGAGCTAGAGAAAACGGAAGATA TTACG-MGBNFQ	
*Borrelia* spp.	23S rRNA	Bo_bu_sl_23S_F	GAGTCTTAAAAGGGCGATTTAGT	[24]
		Bo_bu_sl_23S_R	CTTCAGCCTGGCCATAAATAG	
		Bo_bu_sl_23S_P	FAM-AGATGTGGTAGACCCGAAGCCGAGT-BHQ1	
*Ehrlichia chaffeensis*	*Dsb*	Eh_ch_dsb_F	TATTGCTAATTACCCTCAAAAAGTC	[24]
		Eh_ch_dsb_R	GAGCTATCCTCAAGTTCAGATTT	
		Eh_ch_dsb_P	FAM-ATTGACCTCCTAACTAGAGGGCAAGCA-BHQ1	
*Ehrlichia ewingii*	16S rRNA	Modified	CATGCAAGTCGAACGAACAATTCCTA	[31], Modified for this study
		Modified	TCCATACTACTAGGTAGATTCCTACGCAT	
		Modified	FAM-TCTCTGACTATTTAGATAGTTGTTAGTGGCA-MGBNFQ	
*Ehrlichia muris eauclairensis*	omp p13	p13-116-F	AAAGAGTCAGTGTTGATCCG	[32]
		p13-196-R	CAATGATGATACTGCGAACA	
		p13-147-FAM	FAM-TTGCTATTGGCTGGGTTTTTT AGT-MGBNFQ	
*Rickettsia amblyommatis*	ompB	Ra_F	GGTGCTGCGGCTTCTACATTAG	[33]
		Ra_R	CTGAAACTTGAATAAATCCATTAGTAACAT	
		Ra_P	FAM-TCCTCTTACACTTGGACAGAATGCT-MGBNFQ	
*Rickettsia montanensis*	ompB	RMF2832	GCGGTGGTGTTCCTAATACRM	[34]
		R2937	CCTAAGTTGTTATAGTCTGTAGTG	
		RMB2875	FAM-CAAAGATGCTAGCGCTTCACAGTTA-MGBNFQ	Modified for this study
*Rickettsia parkeri*	ompB	Rp_F	CAAATGTTGCAGTTCCTCTAAATG	[35]
		Rp_R	AAAACAAACCGTTAAAACTACCG	
		Rp_P	FAM-AAATTAATACCCTTATGAGCAGCA-MGBNFQ	Modified for this study
*Rickettsia philipii*	*nusG*	nusG_F.124	AGGATGTCATTGTTGCGGGA	[36]
		nusG_R.186	TCTCCTAGATTGCCACGCAG	
		Probe nusG_P_FAM	FAM-ATGCGTCGTCATAGCCGTAG-MGBNFQ	
*Rickettsia rickettsii*	hypothetical protein gene	Rr_F	AAATCAACGGAAGAGCAAAAC	[35]
		Rr_R	CCCTCCACTACCTGCATCAT	
		Rr_P	FAM-TCCTCTCCAATCAGCGATTC-MGBNFQ	

## Results

### Tick species submissions

**Fig 1:**
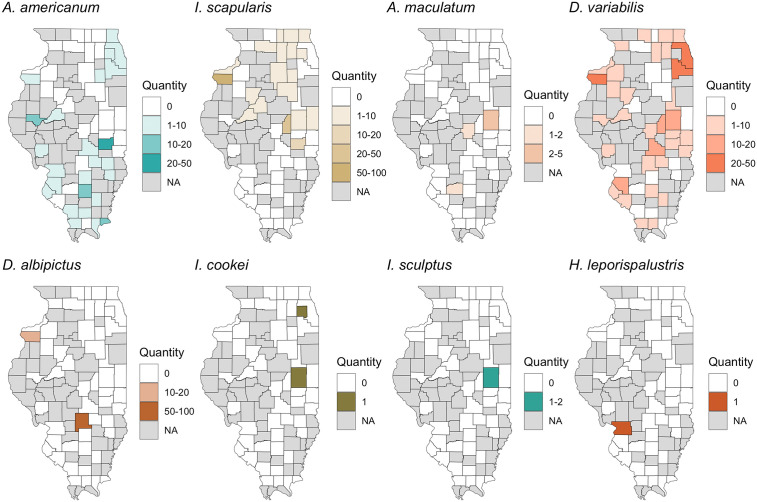
Overview of community-submitted tick samples between 2018-2023 by species and county. Ticks where a history of travel was indicated or where identification was not possible beyond the genus level are not included.

Ticks were submitted to the program between 2018 and 2023 from a total of 54 counties. Submissions from Illinois residents indicating travel or a likely place of tick encounter in neighboring states (including Indiana and Wisconsin) were excluded from the dataset. In total, 740 tick specimens were submitted (82, 208, 119, 121, 84, 126, per year for 2018–2023, respectively). Eight different species of ticks were collected as part of our passive tick surveillance program. The most common, in terms of numbers of specimens, were *D. variabilis* (338), *A. americanum* (146), *I. scapularis* (150), as well as *D. albipictus* (82). Additionally, through this program we detected 6 *A. maculatum* (3 females and 3 males) in three counties, as well as a small number of *I. cookei* (2 females), *I. sculptus* (2), and *H. leporispalustris* (1). The distribution by county for these submissions (excluding submissions with a history of travel in the past ten days) is provided in Fig 1. The majority of specimens were adults (85.4%), with the remainder split among larvae (8.9%) and nymphs (5.7%). Similar patterns were observed at the species level, with 97.3% of *I. scapularis* (101 females and 45 males), and 99.7% of *D. variabilis* being adults (170 females and 167 males). For *A. americanum*, there was a higher proportion of juvenile ticks among the submissions, with 36.9% adults (28 females and 26 males), 17.8% nymphs, and 45.2% larvae (Table 2).

**Table 2:** Overview of total number of ticks by species and life stage submitted and identified as part of the passive surveillance program between 2018-2023.

Species	Stage	Totals	Origin unclear*	Years detected
*Amblyomma americanum*	Adult	54	3	5/6
	Larva	66	0	1/6
	Nymph	26	4	4/6
*Amblyomma maculatum*	Adult	6	1	4/6
*Amblyomma* spp	Nymph	5	4	1/6
*Dermacentor albipictus*	Adult	82	0	3/6
*Dermacentor* spp.	Adult	1	1	1/6
*Dermacentor variabilis*	Adult	337	11	6/6
	Nymph	1	1	1/6
*Haemaphysalis leporispalustris*	Nymph	1	0	1/6
*Ixodes cookei*	Adult	2	0	2/6
*Ixodes scapularis*	Adult	146	1	6/6
	Nymph	4	0	2/6
*Ixodes sculptus*	Nymph	2	0	1/6
*Ixodes* spp	Adult	4	0	3/6
*Ixodes* spp	Nymph	2	0	1/6

*Indicates the number of specimens per species and stage whereby travel was indicated or where the county of origin was unclear (e.g., multiple or no counties provided).

**Fig 2:**
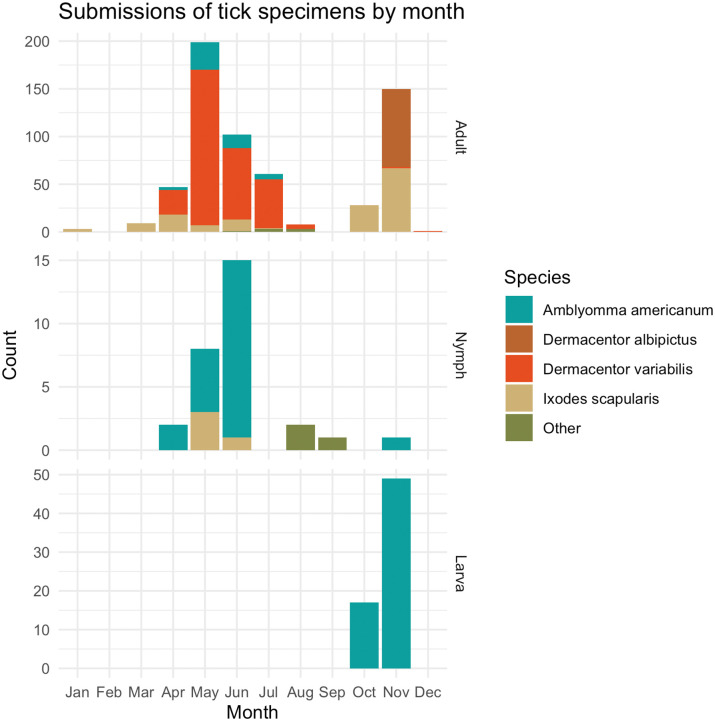
Submissions of tick specimens by species and month of collection between 2018-2023 based on indicated encounter date. The “Other” category includes *A. maculatum*, *I. cookei*, *I. sculptus*, and *H. leporispalustris*.

The largest number of ticks were submitted in the months of May and June during the six-year period. The majority of submissions during this period were *D. variabilis* and *A. americanum* (Fig 2). Another peak in submissions occurred in the autumn months of October and November, which consisted mainly of *I. scapularis*, as well as submissions of *D. albipictus*, and a number of larval *A. americanum*. The broadest range of activity was found in *I. scapularis*, with submissions occurring during 8 months of the year, emphasizing the almost year-round potential for human exposure to this vector and its associated pathogens.

Submissions of tick specimens indicated associations with a range of host species (Table 3). The most common hosts on which ticks were found were humans (33.1%), followed by deer (*Odocoileus virginianus*)(16.1%) and dogs (*Canis familiaris*)(15.8%). Other hosts included unspecified farm animals, cats (*Felis catus*), rabbits (species not known), raccoons (*Procyon lotor*), woodchucks (*Marmota monax*), and one submission from an indigo bunting (*Passerina cyanea*). A considerable proportion (28.4%) of submissions did not indicate the host type that the tick was found on. The host distributions among species highlights that *A. americanum* and *I. scapularis* had broad host ranges but were most commonly submitted after detection on dogs. In contrast, the largest proportion of submissions of *D. variabilis* came from humans. Submitted *A. maculatum* were associated with detections on humans, while those of *D. albipictus* were all associated with deer. We also detected two *I. sculptus* that were attached to a woodchuck. Likewise, we identified an *I. cookei* that had been attached to a woodchuck, and another that was found attached to a human.

**Table 3:** Distribution of submitted tick specimens by species and indicated type of host on which the specimen was found. Values in brackets indicate the relative proportion among hosts for each tick species.

Species	Unknown	Dog	Environment	Farm animals	Human	Deer	Cat	Rabbit	Raccoon	Woodchuck	Indigo Bunting
*A. americanum*	40 (0.27)	53 (0.36)	1 (0.007)	14 (0.09)	38 (0.26)	0	0	0	0	0	0
*A. maculatum*	2 (0.33)	0	0	0	4 (0.67)	0	0	0	0	0	0
*Amblyomma* spp	5 (1)	0	0	0	0	0	0	0	0	0	0
*D. albipictus*	0	0	0	0	0	82 (1)	0	0	0	0	0
*Dermacentor* spp.	1 (1)	0	0	0	0	0	0	0	0	0	0
*D. variabilis*	123 (0.36)	15 (0.04)	0	2 (0.006)	185 (0.55)	0	3 (0.009)	1 0.003)	9 (0.03)	0	0
*H. leporispalustris*	1 (1)	0	0	0	0	0	0	0	0	0	0
*I. cookei*	0	0	0	0	1 (0.5)	0	0	0	0	1 (0.5)	0
*I. scapularis*	35 (0.23)	46 (0.31)	0	0	16 (0.11)	37 (0.24)	4 (0.03)	11 (0.07)	0	0	1 (0.006)
*I. sculptus*	0	0	0	0	0	0	0	0	0	2 (1)	0
*Ixodes* spp	2 (0.33)	3 (0.5)	0	0	1 (0.17)	0	0	0	0	0	0

### Pathogen testing

**Fig 3:**
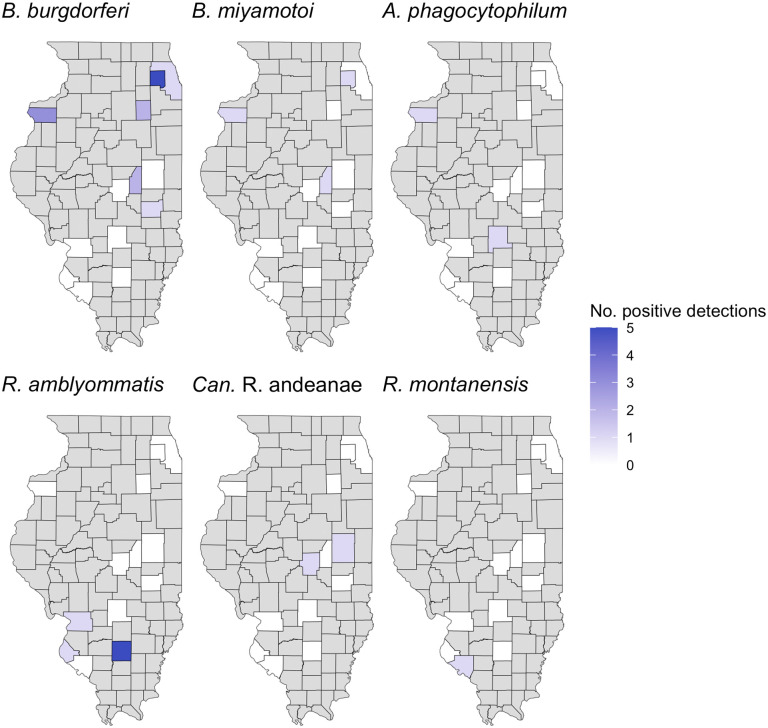
Geographic distribution of positive samples among tested ticks for *B. burgdorferi* s.s., *B. miyamotoi*, *A. phagocytophilum*, *R. amblyommatis*, *Candidatus* R. andeanae and *R. montanensis.* No ticks were tested in this study from counties that are colored grey.

We obtained pathogen testing results for a total of 169 ticks in 161 pools for which a county was indicated and no travel within the past ten days was indicated (ca. 22.5% of submitted ticks). The geographic distribution of positive detections is presented in Fig 3. Detected pathogens included *B. burgdorferi* s.s., which was found in ticks from six counties. Among *I. scapularis* (85 adults) the overall prevalence was 14% [CI: 8–23%] (Table 4). The prevalence for *B. miyamotoi,* which was detected in 3 counties in *I. scapularis*, was 4.7% [CI: 1.5–11%]. Within adult *I. scapularis*, the prevalence for *A. phagocytophilum* was 1.2% [CI: 0.06–5%]. However, this pathogen was also detected in a *D. albipictus* male from Fayette County. Two *I. scapularis* specimens were coinfected with *B. burgdorferi* s.s. and *B. miyamotoi*, and *B. burgdorferi* s.s. and *A. phagocytophilum*, respectively.

**Table 4:** Pathogen detections and prevalence estimates (proportion infected) by tick species and stage. Values in parentheses indicate confidence intervals.

Species	Stage	No. tested	*B. burgdorferi s.s.*	*B. miyamotoi*	*A. phagocytophilum*	*R. amblyommatis*	*R. montanensis*	*C.* R. andeanae
*A. americanum*	Adult	4	–	–	–	0.25 (0.016-0.72)	–	–
*A. americanum*	Nymph	14	–	–	–	0.53 (0.18-0.89)	–	–
*Amblyomma* sp.	Nymph	1	–	–	–	1 (0.14-1)	–	–
*A. maculatum*	Adult	3	–	–	–	–	–	0.66 (0.16-0.97)
*D. albipictus*	Adult	7	–	–	0.14 (0.008-0.49)	–	–	–
*D. variabilis*	Adult	48	–	–	–	–	0.021 (0.001-0.09)	–
*H. leporispalustris*	Nymph	1	–	–	–	1 (0.14-1)	–	–
*I. scapularis*	Adult	85	0.14 (0.08-0.23)	0.047 (0.015-0.11)	0.012 (0.0006-0.05)	–	–	–
*Ixodes* sp.	Adult	4	0.5 (0.11-0.89)	–	–	–	–	–
*Ixodes* sp.	Nymph	2	–	–	–	–	–	–

The other pathogens that were detected from these samples included three spotted fever *Rickettsia* species. This included *R. amblyommatis*, which was primarily found in *A. americanum*, as well as is in a *H. leporispalustris*. The prevalence in nymphs of *A. americanum* was 53% [CI: 18–89%]. One out of the four adult *A. americanum* was positive for *R. amblyommatis*. Through sequencing of samples, we additionally detected *Candidatus* R. andeanae in two *A. maculatum* specimens, as well as *R. montanensis* in a *D. variabilis*.

Tests for *B. microti*, *E. muris eauclairensis*, *E. chaffeensis*, *E. ewingii*, *R. parkeri*, *R. rickettsii*, and *R. philipii* and *Bartonella* sp. (the latter two tested for a subset of the samples) were all negative.

## Discussion

Through a multi-year community-engaged passive tick surveillance program that has been maintained at the Illinois Natural History Survey Medical Entomology Lab we documented a diversity of tick species that Illinois residents encountered. Through testing a subset of these specimens for a range of tick-borne pathogens, we also documented the presence of six pathogens in different tick species, and the presence of coinfections in *I. scapularis*. This work adds to our picture of risk of exposure to ticks and tick-borne pathogens present in this region. Although peaks in tick collections and submissions occurred during May-June and October-November, ticks were collected by participants in all months of the year except for February, highlighting that some level of risk of exposure is present throughout the year.

The majority of specimens that were submitted were adult ticks, which is likely a reflection of the greater likelihood of people detecting adult ticks on themselves or animals, due to the relative size of this life stage over nymphs or larvae. That the most-commonly submitted species was *D. variabilis* likewise might reflect their abundance, as well as the large size of the adults of this species. Higher rates of submissions to passive surveillance programs of adult specimens has been documented previously [37,38], although not as pronounced as in our study. It is an open question whether this reflects the ongoing expansion of ticks in Illinois and possibly a lower level of awareness or prior exposure to tick bites, which has been shown to be associated with performing skin checks for ticks [39].

The diversity of hosts from which specimens were submitted adds to our knowledge of tick-host associations in the state [23], but also reflects the diversity of stakeholders and community participation in this program. This included samples provided by employees of Forest Preserve Districts and Park Districts, researchers, hunters, and members of the general public. This shows how engagement of a broad base of participants in the program helps cast a broad net resulting in collections from less common hosts, as well as rarer tick species, or collections from locations where finding suitable dragging sites may be challenging. At the same time, a caveat to the current findings is that due to the relatively small-scale nature of the program, and that recruitment largely occurred through word-of-mouth, personal interactions, social media engagement with local groups associated with outdoor activities, and through tickborne disease educational and outreach efforts, the distribution of ticks and pathogens found as part of this study should be seen as additional opportunities for discovery of presence of ticks and pathogens in counties, rather than as comprehensive representations of statewide distribution patterns.

A number of the tick-host associations were notable. For instance, this work highlights that *A. maculatum*, a species that has been expanding its range in the state [11,40,41] is also detected on humans, emphasizing the risk for exposure to pathogens associated with it. We also detected an *I. cookei*, the groundhog tick, which had been attached to a human. This species is a main vector of Powassan virus, which can cause serious illness in humans [42,43]. This finding highlights a particular utility of passive collections in the detection of potential vectors that are rarely collected by means of dragging, and is in line with previous studies on human tick encounters which have frequently documented encounters with these types of vectors, including *D. albipictus,* and *I. cookei* [44].

The pathogens that were detected in this study were composed of those typically associated with *I. scapularis*, including *B. burgdorferi s.s.*, and SFGR species. The most common pathogen in *I. scapularis* was *B. burgdorferi s.s.*. The estimated prevalence of infection with *B. burgdorferi s.s.* was, however, relatively low (14%) when compared to estimates from other locations, or from active surveillance in Illinois where estimates were approximately 39% for adult females (C. Stone, personal communication). This relatively low prevalence could reflect the variable condition of ticks that were submitted, with the possibility that degradation of DNA in some samples could have resulted in an underestimate of true prevalence. Additionally, as these results include ticks that were unfed, as well as engorged specimens that had been attached to a variety of host species (which may also have affected amplification of DNA [45]), care should be taken with interpretation of the prevalence rates.

We also detected the presence of *B. miyamotoi* and *A. phagocytophilum* in *I. scapularis*,. Additionally, *A. phagocytophilum* was found in a single *D. albipictus* that had been associated with a white-tailed deer. Previous work has detected high rates of infection of *A*. *phagocytophilum* in *D.* *albipictus* collected from white-tailed deer in Minnesota [46], but those were most similar to a presumed non-pathogenic variant. We did not sequence to distinguish these variants, but it is likely that this was the case here as well. This leads to the possibility that multiple strains of *A. phagocytophilum* are present in Illinois, which should be investigated through additional targeted surveillance efforts and sequencing.

Other pathogens that were detected were all SFG rickettsial species where the level of human pathogenicity remains unclear. These included *R. amblyommatis*, which was present in a significant proportion of *A. americanum*, comparable to findings based on drag and trap collections of *A. americanum* in Illinois [41]. Sequencing also confirmed the presence of this pathogen in an *H. leporispalustris* specimen (accession number PZ482915). This species, the rabbit tick, is primarily associated with rabbits and hares, other small mammals, and birds, but is thought to attach only very rarely to humans [47]. A number of *Rickettsia* sp. have been detected in *H. leporispalustrus*, including *R. rickettsia* [48], *R. felis* [49], *R. lanei* [50,51], and *Rickettsia* sp. ME2023 [52]. An area for further research is thus whether and how *H. leporispalustris* contributes to the maintenance of various SFGR species in Illinois.

Through sequencing, we also detected *R. montanensis* in *D. variabilis* in the state, an SFGR that has been shown to cause mild infection in guinea pig models [53], as well as *Candidatus* R. andeanae, a species of unknown pathogenicity, in *A. maculatum*. The same *Rickettsia* sp. were detected in samples from active surveillance [41], and adds to the evidence base for the presence of a range of SFGR species present in southern and central Illinois, but limited evidence for the presence of *R. rickettsii*.

The detection of less common pathogens, or of tick-borne pathogens in species that are not typically associated with that pathogen, as we documented instances of in this study, points to one of the advantages of a high-throughput assay such as the Fluidigm method we used here, and has been used for tick surveillance in other locations [24]. A downside is that the cost per specimen tested is relatively high (in our case, ca. $48 per sample, excluding the costs of extractions and consumables), and although there is ample capacity to include assays for additional pathogens, the cost of positive controls and probes does become relevant for very broad panels. For this reason, when results were inconclusive (e.g., the cross-reactivity we observed between *R. amblyommatis* and *R. montanensis*), follow-up rt-PCR testing and/or sequencing for confirmations was the more cost-effective approach versus additional runs of the Fluidigm system to optimize the current suite of assays. Additional optimization and expansion of the pathogen panel, particularly for tick-borne viruses such as Heartland virus (which we have previously detected in Illinois [12]) and Powassan virus, is planned for future pathogen testing of samples from our ongoing passive surveillance program.

Future work related to passive surveillance in this region that we are developing will focus on expanding the collaborations with stakeholders to cast a broad net to enhance capacity for detections of invasive species such as *H. longicornis*, which has recently been detected in Illinois, and which has benefited from passive surveillance efforts in other regions [54]. Further, there is scope to use such programs to learn more about the demographics of people at risk (e.g., [55,56]) in the state as well as protective behaviors they engage in, and the types of habitats where they or their family members report exposure to tick bites. Further research, with a longer-term and larger-scale passive dataset, could explore how submission propensity varies by county and month in relation to population size, as well as outreach events or news articles that may influence tickborne disease awareness. Additionally, this type of broad survey of ticks and pathogens can inform targeted active surveillance to develop estimates of risk of exposure, for instance related to rare or emerging pathogens or tick-host exposures that might be picked up through passive collections.
